# Fungal Diversity in the Soil of the *Oxytropis glacialis* Root System on the Qinghai-Tibet Plateau

**DOI:** 10.3389/fmicb.2022.831783

**Published:** 2022-02-24

**Authors:** Peng-xi Cao, Yixuan Liu, Hong-mei Ma, Ning Zhao, Shu-ting Chen, Guo-qi Xu, Xing Liu

**Affiliations:** ^1^Ecological Field Station Real-Time Monitoring Center, Research Center for Ecology, Tibet University, Lhasa, China; ^2^Laboratory of Adaptation and Evolution of Plateau Biota to Extreme Environments, Research Center for Ecology and Environment of Qinghai-Tibetan Plateau, Tibet University, Lhasa, China; ^3^College of Life Science, Wuhan University, Wuhan, China

**Keywords:** core microbiota, *Oxytropis glacialis*, QTP, soil fungal of root system, swainonine-producing fungal

## Abstract

Because of swainonine-producing endophytic fungal, *Oxytropis glacialis* is one of the main poisonous weeds in the alpine grassland and desert grassland of the Qinghai-Tibet Plateau (QTP). It has a severe impact on grassland degradation on the QTP. In this manuscript, the Internally Transcribed Spacer (ITS) region of fungal communities in the soil of the *O. glacialis* root system was sequenced by high-throughput sequencing and analyzed by bioinformatics methods. The physical and chemical properties of the soil samples were analyzed in combination with the fungal diversity and its relationship with the soil physical and chemical factors. The results showed that the soil fungal community in the *O. glacialis* root system are rich in diversity in different ecological environments and are most affected by the soil pH value and organic matter. The swainonine-producing fungal *Embellisia oxytropis* was first detected in the soil of the *O. glacialis* root system. This finding provides data to support the next step in demonstrating the horizontal spread of swainone-producing fungal from *O. glacialis* to soil. In addition, a stable network of core flora has a facilitating effect on the formation of *O. glacialis* as a dominant species in alpine ecosystems.

## Introduction

The Qinghai-Tibet Plateau (QTP) is commonly called the third pole in the world. Its ecological type is unique. Biodiversity bred in extreme environments is extremely rich and has special biological resources (Xiao et al., 2003). To adapt to this extreme environment, root microorganisms and plants have constructed a complex and stable interaction system, reflecting the adaptation strategies of microorganisms and plants (Hong et al., 2018). According to statistics, the alpine grassland area of the QTP accounts for 37.64% of the national grassland; out of this area, the alpine degraded grassland area accounts for nearly 1/3 of the QTP grassland area, which is as much as 425,000 Km^2^ (Zhou et al., 2005; Tian et al., 2009). In recent years, the alpine grasslands on the QTP have been degraded more seriously. Locoweeds are an important part of the alpine grassland ecosystem. They are mainly distributed in alpine degraded grasslands, alpine grasslands, alpine saline deserts, and alpine deserts with high soil sand. The expansion rate of locoweed is consistent with the degradation rate of natural grassland, and some areas or local environments have an irreversible trend (Hu et al., 2011; Chen et al., 2012). Among more than 20 locoweeds of *Oxytropis* in China, 11 species are distributed in the alpine grasslands of the Tibetan Plateau, including *Oxytropis glacialis*, *Oxytropis ochrocephala*, *Oxytropis sericopetala*, *Oxytropis glabra*, and *Oxytropis falcate* (Zhao et al., 2004). These species can quickly form dominant species or subdominant species in their habitats or local areas, and they pose a serious threat to the stability of the ecological environment. *O. glacialis* is one of the poisonous plants and is the most widely distributed and unique Madagascar genus of alpine grasslands on the Tibetan Plateau (Quan et al., 2009).

*Oxytropis glacialis* is a perennial herb with extremely shortened stems and well-developed root systems. It grows on hillside gravel, riverbeach gravel, sandy land, and other places at an altitude of 4500–5400 m. These plants are widely distributed in Tibetan Gaize, Bangor, Coqin, Geji, Zhongba, Tingri, Nima, Shenza, Shuanghu, Ritu, Pulan, Jilong, Sakya, and other places. The whole plant of *O. glacialis* is poisonous. It contains a toxic alkaloid swainonine (SW, 1,2,8-trihydroxyoctahydro indolizidine), which is highly toxic to animals and can cause dysfunction of the central nervous system of animals (Guo, 2005). Locoweed species that contain SW, produced by an endophytic fungal called *Embellisia oxytropis* rather than *Oxytropis* itself, have a strong ecotoxicological effect and play a key role in the degradation of alpine grasslands. In addition, SW-producing fungal play a certain role in many hosts and habitats (Yu et al., 2009). *E. oxytropis* can be isolated and purified from plant roots, stems, leaves, petioles, flowers, pods, and seeds (Cook et al., 2011), and its propagation occurs through vertical transmission of seeds (maternal transmission) or horizontal transmission between infected plants (Ralphs et al., 2008; Huang and Huang, 2012). Most of the current research on *O. glacialis* focuses on its ecological toxicology, the chemical properties and biological toxicity of SW, the life history of *E. oxytropis*, and the diversity of fungal in its root soil or whether the root soil contains SW; nevertheless, no research has been conducted on the horizontal transmission and transmission routes of fungal or swainonine (SW) fungal from root soil.

This study takes the soil fungal of *O. glacialis* roots from different ecological environments on the QTP as the research object. This research explores the spatial distribution, community diversity, and structure of the soil fungal of *O. glacialis* roots in different ecological environments, as well as its relationship with the soil physical and chemical factors to explore the possible impact of fungal community on the formation of dominant species of *O. glacialis* in alpine ecosystems. Besides, this study provides a reference for grassland degradation and desertification control in the QTP.

## Materials and Methods

### Sampling Area Summary

Samples were taken from four locations (Table 1): the Yangbajing Basin, which is rich in geothermal resources; the core experimental area of the Everest National Nature Reserve; and Zarinanmucuo and Zabuyecuo, which are located in Qiangtang National Nature Reserve in the northern Tibetan Plateau. This scientific investigation has been reported to and approved by the Tibet Autonomous Region Forestry Department and the competent departments of nature reserves.

**TABLE 1:** Sample site information.

Location	Samples	Longitude (E)	Latitude (N)	Altitude/(m)	Ecotype
Yangbajing Town, Dangxiong County	YBJ	90.48799°	30.04268°	4,287	Alpine depleted range
Everest Base Camp, Dingri County	ZF	86.84309°	28.16793°	5,003	Alpine desert steppe
Zabuyecho, Zhongba County	ZBY	84.02377°	31.39431°	4,453	Alpine salt desert
Zharinanmucuo, Couqin County	CQ	86.04237°	31.03991°	4,710	Alpine steppe

### Plot Setting and Sampling Method

This research started in August 2017, and *O. glacialis* was sampled during the peak period. There were a total of four sampling points (Table 1). Each sample point was set with three repeats, and the distance between the three repeats was above 100 m (Dombrowski et al., 2017). A total of 12 samples were collected. In particular, soil of root system, also called root-zone soil, refers to soil that is loosely attached to or close to the roots, while bulk soil is soil outside the root zone, far from the plant’s roots (Derr et al., 2016). For the sampling, the whole plant of *O. glacialis* was dug up, the bulk soil was shaken into a sterile bag for the determination of the soil physical and chemical factors, and then the soil of root system of the plants was rinsed into 95% ethanol and loaded into sterile tubes. Then, the samples were stored at −20°C in a car refrigerator (Meigu Mobicool, CF-50), brought back to the laboratory, and placed in a −80°C ultralow temperature refrigerator (Jiangsu Shenglan, DW86L-158) for cryopreservation. Finally, the samples were used for total DNA extraction of soil fungal microbiome.

### Analysis of the Soil Physical and Chemical Properties

The environmental factors to be measured included pH value, soil moisture (SM), electrical conductivity (EC), organic matter (OM), available K (AK), available P (AP), and ammonium N (AN), for a total of seven indicators. The pH was measured by the potentiometer method (HANA, HI98103), and the ratio of water and soil was 1:1. The soil water content was measured by the high-temperature drying method. Five grams of fresh soil was weighed and placed in a constant-temperature drying box (Shanghai Jinghong, DK-420S) and measured at 105 ± 2°C for 48 h. The calculation formula was SM (%) = (5-dry weight)/5 × 100%. The conductivity was determined by the conductivity meter method (HANA, HI98304), and the water-soil ratio was 3:1. OM, available potassium, available phosphorus, and ammonium nitrogen were measured by a portable soil composition analyzer (Beijing Youputongyong, UPA–B506); OM measurement was based on the potassium complicate method, available potassium was based on ammonium acetate extraction-atomic absorption spectrophotometry, available phosphorus was based on sodium bicarbonate extraction-molybdenum antimony anti-spectrophotometry, and ammonium nitrogen is based on Nessler reagent colorimetry (Cao et al., 2019).

### Extraction and Sequencing of Soil Fungal Total DNA From the Root System

The total DNA of the soil fungal of root system was extracted by the sodium dodecyl sulfate (SDS) plus enzyme method (Liu et al., 2015). The primers were ITS1F (5′-CTTGGTCATTTAGAGGAAGTAA-3′) and ITS2R (5′-GCTGCGTTCTTCATCGATGC-3′) for PCR amplification of the fungal ITS1-ITS2 region (Adams et al., 2013). PCR used a 20 μl reaction system: 10X buffer, 2 μl; 2.5 mM dNTPs, 2 μl; bidirectional primers (5 μM), 0.8 μl; rTaq polymerase, 0.2 μl; BSA, 0.2 μl; template, DNA 10 ng; and ddH2O supplement to 20 μl. The PCR parameters were 95°C for 3 min, followed by 25 cycles of 95°C for 30 s, 56°C for 30 s, and 72°C for 45 s, then 72°C for 10 min, and 10°C until halted by user. NanoDrop One was used to detect the purity and concentration of PCR products, and the samples were sent to Shanghai Majorbio Biopharm Technology Co., Ltd. for sequencing using the Illumina HiSeq platform.

### Data Analysis

Using Illumina HiSeq sequencing to obtain paired-end double-end sequence splicing with FLAS software (FLAS 1.2.11), the raw tags obtained by Trimmomatic software were subjected to sequence defiltering to obtain high-quality clean tag data. Usearch software was used to perform sequence classification and annotation on clean tag data in the Silva database base, and clustering and species classification statistical analysis were performed based on the operational classification unit OTU (operational taxonomic unit) data of 97% similarity pairs (Nouioui et al., 2018). Analysis of similarities (ANOSIM) similarity analysis based on the minimum number of samples was used to determine whether the sample grouping setting is feasible. Mothur software (version v.1.30.1) was used to analyze the alpha diversity, including the Shannon, Sobs, Simpson, Ace, Chao, and coverage indexes, and a *t*-test was used to test the difference between the diversity groups. Beta diversity was analyzed through non-metric multidimensional scaling (NMDS) non-metric multidimensional scaling analysis. The bar graph and Venn diagram reflect the structure and function of fungal community. Different species and functional genes of different fungal community, using One_way ANOVA single factor analysis of variance, Kruskal-Wallis *H* test multiple test correction Fdr, *Post hoc* test Tukey-kramer level value is 0.95, and passed Linear discriminant analysis Effect Size (LEfSe) Discriminant analysis of multilevel species differences determined the species and genes with significantly different enrichment. In terms of fungal structure association and model prediction, random forest analysis was used to determine the ecological evolution process of the fungal community, and the network co-occurrence network and the correlation network were used to determine the core flora and key species. Using MEGA software, the selected SW-producing fungal sequences were combined with known related fungal sequences to construct an NJ phylogenetic tree. Variance expansion factor (VIF) was used to screen environmental factors and select environmental factors that have a greater effect on the flora, analyze the impact of environmental factors on the flora through canonical correspondence analysis (CCA) and use the correlation heatmap based on the Spearman coefficient to analyze the diversity of environmental factors on the flora and the specific impact of sexuality and structural function. The original pictures of related SVGs were grouped and processed by PS (Adobe Photoshop CS6) and AI (Adobe Illustrator CS6).

The raw sequencing read dataset was deposited in the National Center for Biotechnology Information (NCBI) Sequence Read Archive with accession No. PRJNA630993.

## Results

### Sequencing Results and Alpha and Beta Diversity Analysis

#### Sequencing Results

**FIGURE 1:**
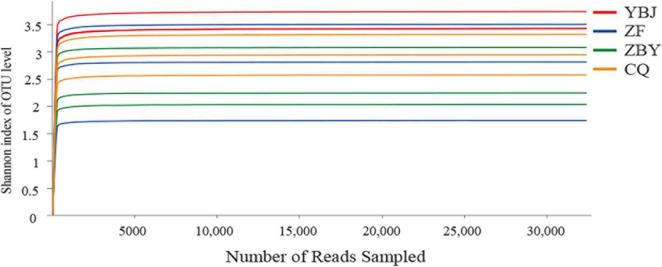
Shannon dilution curve and diversity index difference.

Based on high-throughput sequencing, a total of 711,620 original sequences were obtained, and 388,248 high-quality sequences were obtained after quality control. The average number of reads for each sample was 32,354. The number of OTUs annotated at a similar level of 97% was 1,014, including 5 phyla, 22 classes, 68 orders, 129 families, and 231 genera. The average coverage of the library was 99.91%, and the Shannon dilution curve tended to be flat (Figure 1), indicating that the sequencing data of this study are reasonable. More sequencing data would only generate a small number of new species OTUs, and most of the fungal are included in the library, which can reflect the community structure of fungal in the sample. The *R*-value obtained by ANOSIM similarity analysis was 0.9259, indicating that the difference between the groups was greater than the difference within the group, and the grouping was feasible. The Adonis substitution multivariate analysis of variance analysis showed that the *P*-value was 0.001, and the grouping reliability was high.

### Alpha Diversity Analysis

Analysis of the species richness and diversity indexes of the four groups of samples included the six indexes of Ace, Chao, Coverage, Shannon, Simpson, and Sobs. Table 2 shows that the species richness and diversity of the soil fungal in the roots of *O. glacialis* in the four regions were ranked as YBJ > CQ > ZF > ZBY. The richness index of Ace, Chao, and Sobs was the highest for YBJ and the lowest for ZBY. The Shannon diversity index is the highest for YBJ and the lowest for ZBY. The Simpson diversity index is the highest for ZBY and the lowest for YBJ; these findings are consistent with the performance of the Shannon index. The Coverage index table is between 99.85 and 99.97%; the ZBY has the highest coverage, while the YBJ has the lowest coverage.

**TABLE 2:** The species richness and diversity index of soil fungal in the *Oxytropis glacialis* root system.

Samples	Sequences	OTUs	Alpha diversity					
			
			Ace	Chao	Coverage	Shannon	Simpson	Sobs
YBJ	53637	312	344	342.69	99.85%	3.53	0.06610	312
ZF	63856	161	178	182.11	99.92%	2.69	0.16196	161
ZBY	60764	123	128	129.94	99.97%	2.45	0.23022	123
CQ	58949	231	260	262.99	99.88%	2.95	0.16423	231

### Analysis of the Species Composition of the Soil Fungal Community in the *Oxytropis glacialis* Root System

#### Community Structure of Fungal Community at the Phylum and Genus Levels

**FIGURE 2:**
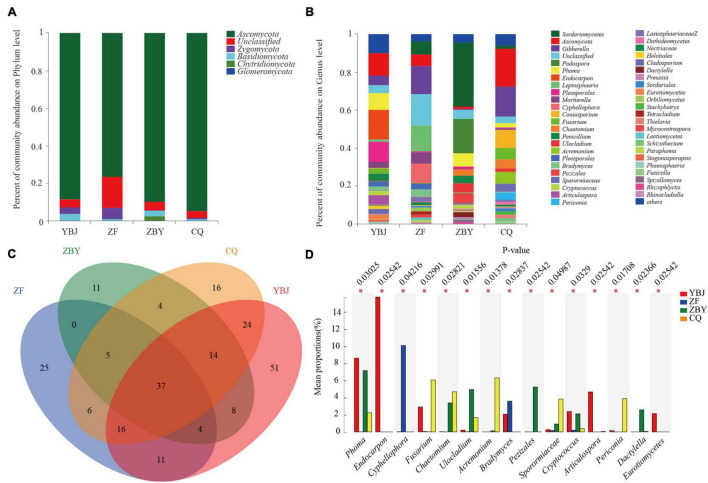
Root system soil fungal community structure at the genus and phylum levels and a Venn diagram based on the genus level. **(A,B)** Fungal community structure based on the phylum and genus levels. **(C)** Venn diagram based on the genus level. **(D)** Analysis of differences between groups of fungal communities (*0.01 < *P* ≤ 0.05).

According to the taxonomic analysis results, samples were annotated to a total of 5 phyla (Figure 2A), including Ascomycota (87.24%), Zygomycota (2.65%), Basidiomycota (1.99%), Chytridiomycota (0.83%), and Glomeromycota (0.01%), except for 7.29% that were unclassified. According to the statistics, the four groups of samples all contained more than five annotated fungal. Ascomycota was the main dominant entry for the YBJ (88.37%), ZF (76.32%), ZBY (89.63%), and CQ (94.63%) samples. The samples for YBJ (4.3%), ZF (16.61%), ZBY (4.7%), and CQ (3.54%) all accounted for a large proportion, indicating that there are abundant unknown potential taxa for the root fungal of *O. glacialis*. The average relative abundance of the four groups of samples was in the top 1%. The YBJ samples contained Zygomycota (3.48%) and Basidiomycetes (3.51%), the ZF samples contained Zygomycota (6.01%), the ZBY samples contained Basidiomycetes phyla (2.89%) and Phytophthora (2.54%), and the CQ samples had no average relative abundance in the top 1%.

The results of the genus level analysis (Figure 2B) show that all samples were annotated to 232 genera and 7.29% were undetermined taxa. The average relative abundance of the top 1% of the species was distributed in 20 genera of 3 phyla, accounting for 64.62% of all genera. Based on the genus level, a Venn diagram was used to count the number of common and unique genera in the four groups of samples (Figure 2C). There were 37 genera (15.95%) in four groups, 39 (16.81%) in three groups, 53 (22.84%) in two groups, and 103 (44.4%) in a single group, including 51 (21.30%) endemic genera in the YBJ sample, 25 (10.78%) endemic genera in the ZF sample, 11 (4.74%) endemic genera in the ZBY sample, and 16 (6.9%) endemic genera in the CQ sample. At the genus level, in at least two samples, the total genera accounted for 55.6%, and the endemic genera accounted for 44.4%. Statistics show that YBJ, ZF, ZBY, and CQ have 165, 104, 83, and 122 genera, respectively. The YBJ samples contain *Endocarpon* (15.72%), *Ascomycota* (11.99%), and *Pleosporales* (10.49%), the ZF samples contain *Gibberella* (14.76%), *Leptosphaeria* (13.63%), and *Cyphellophora* (10.08%), the ZBY samples mainly contain *Sordariomycetes* (33.60%) and *Podospora* (18.01%), and the CQ samples mainly contained *Ascomycota* (19.92%), *Gibberella* (15.70%), and *Coniosporium* (9.52%). The YBJ endemic species are *Endocarpo*, *Trichoderma*, and *Bovista*, the ZF endemic species are *Orbiliomycetes*, *Rhinocladiella*, and *Spizellomycetales*, the ZBY endemic species are *Rotiferophthora*, and the CQ endemic species are *Amphisphaeriaceae* and *Ascobolaceae*.

### Analysis of the Differences in the Soil Fungal of Root System Communities Between Different Groups

At the genus level, one-way analysis of variance (one-way ANOVA) was used to test the significance of the difference between the four groups of differential species. Then, the *post hoc* test was performed for the species with differences, resulting in the top 15 different species based on relative average abundance (Figure 2D), and four samples have significant differences (*P* < 0.01) in *Phoma*, *Endocarpon*, *Cyphellophora*, *Fusarium*, *Chaetomium*, *Ulocladium*, *Acremonium*, *Bradymyces*, *Pezizales*, *Sporormiaceae*, *Cryptococcus*, *Articulospora*, *Periconia*, *Dactylella*, and *Eurotiomyce* (*P* < 0.01).

### Discriminant Analysis of LEfSe Multilevel Species Differences

**FIGURE 3:**
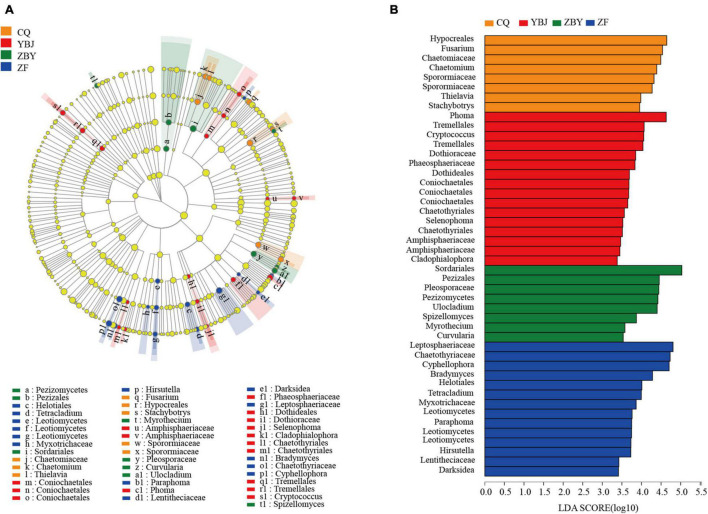
LEfSe multilevel species hierarchy tree diagram and Latent Dirichlet Allocation(LDA) discrimination result diagram. **(A)** LEfSe multilevel species hierarchy tree diagram. **(B)** LDA discriminant result graph. The LDA discriminant column chart counts the microbial groups with significant effects in the four groups. The larger the LDA score is, the greater the impact of species abundance on the difference effect.

To further determine the flora with significant differences in different ecological types (Figure 3A), LEfSe multilevel discriminant analysis (LDA ≥ 2) was used to count from the phylum to genus levels (Figure 3B). For YBJ, approximately eight types of fungal were significantly enriched: *Tremelellas* to *Cryptococcu*, *Coniochaetales*, *Amphisphaeriaceae*, *Phoma*, *Phaeosphaeriaceae*, *Dothideales* to *Selenophoma*, *Cladophialophora*, and *Chaetothyriales*. Nine types of fungal were significantly enriched for ZF, including *Hirsutella*, *Paraphoma*, *Lentitheciaceae* to *Darksidea*, *Leptosphaeriaceae*, *Helotiales* to *Tetracladium, Bradymyces*, *Leotiomycetes*, *Myxotrichaceae*, and *Chaetothyriaceae* to *Cyphellophora*. Five types of fungal were significantly enriched for CQ, including *Chaetomiaceae* to *Chaetomium*, *Thielavia*, *Fusarium*, *Hypocreales* to *Stachybotry*, and *Sporomiaceae*, and six types of fungal were significantly enriched for ZBY, including *Spizellomyces*, *Myrothecium*, *Pezizomycetes* to *Pezizales*, *Sordariales*, *Pleosporaceae* to *Curvularia*, and *Ulocladium*. At the same time, the main groups with a large impact on the difference effect were selected from the four ecological types through LDA linear regression analysis, resulting in YBJ, ZF, CQ, and ZBY containing 16, 14, 8, and 8 groups, respectively. CQ includes Hypocreales, *Fusarium*, Chaetomiaceae, *Chaetomium*, Sporormiaceae, Sporormiaceae, *Thielavia*, *Stachybotrys*. YBJ includes *Phoma*, Tremellales, *Cryptococcus*, Tremellales, Dothioraceae, Phaeosphaeriaceae, Dothideales, Coniochaetales, Coniochaetales Coniochaetales, Chaetothyriales, *Selenophoma*, Chaetothyriales, Amphisphaeriaceae, Amphisphaeriaceae, *Cladophialophora*. ZBY includes Sordariales, Pezizales, Pleosporaceae, Pezizomycetes, *Ulocladium*, Spizellomyces, *Myrothecium*, *Curvularia*. ZF includes Leptosphaeriaceae, Chaetothyriaceae, *Cyphellophora*, Bradymyces, Helotiales, *Tetracladium*, Myxotrichaceae, Leotiomycetes, *Paraphoma*, Leotiomycetes, Leotiomycetes, *Hirsutella*, Lentitheciaceae, Darksidea.

### Correlation and Model Prediction Analysis of Soil Fungal Community Structure in the *Oxytropis glacialis* Root System

#### Co-occurrence Network Analysis

**FIGURE 4:**
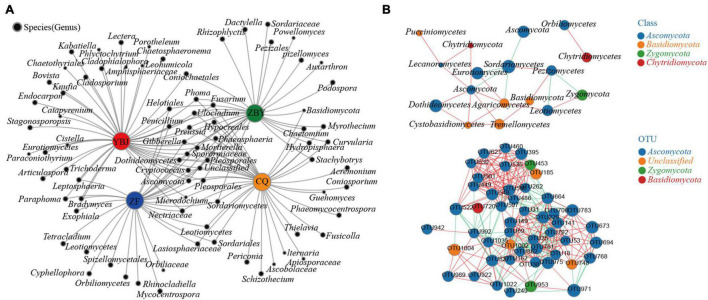
Correlation network of cooccurring and correlation network analysis. **(A)** Collinear network analysis chart based on the genus level. **(B)** Correlation network analysis chart based on 35 phyla, showing species with | SpearmanCoef| > 0.8 and *P* < 0.01.

By selecting all the species with the total abundance in the top 50 for co-occurrence network analysis, the coexistence relationship of the four groups of sample flora in environmental samples was obtained (Figure 4A). The nodes in the network represent the sample node and the species node, and the connection between the species node and the sample node represents that the sample contains this species. All samples and species have a total of 408 effective nodes (Degree). We found that the YBJ, ZF, ZBY, and CQ samples had 142, 83, 79, and 104 nodes, respectively. Approximately 13.36% of the genera that were collinear with the four groups of samples and at least 45.26% belonged to the same line in more than two groups of samples, indicating that the composition of the four groups of samples was quite different. Thirteen species in the four groups of samples had a collinearity rate of more than 1%, including *Sordariomycetes* (10.95%), *Ascomycota* (10.12%), *Gibberella* (9.30%), unclassified (7.53%), *Phoma* (4.65%), *Pleosporales* (4.85%), *Mortierella* (2.74%), *Fusarium* (2.33%), *Chaetomium* (2.09%), *Penicillium* (2.02%), *Ulocladium* (1.77%), *Sporormiaceae* (1.33%), and *Cryptococcus* (1.31%). We identified these 13 species as the core flora of the soil fungal community in the *O. glacialis* root system.

#### Correlation Network Analysis

Correlation network analysis (Figure 4B) revealed that the network contained a total of 17 nodes and 48 edges at the class level, indicating that the soil fungal community of the *O. glacialis* root system had lower connectivity and that the synergy of the overall community was greater than the antagonistic effect. There is greater competition among the colonies, among which the five classes of Ascomycota, Sordariomycetes, Agaricomycetes, Pezizomycetes, and Cystobasidiomycetes are highly correlated and interact with at least the other four classes, which plays a key role in the entire flora network. Ascomycota is the most critical class for the negative correlation of other species; Sordariomycetes is the most critical class for the positive correlation with other species; and Orbiliomycetes and Chytridiomycetes are isolated from the main network and positively related to each other. At the OTU level, the overall positive correlation of the flora with abundance in the top 50 was greater than the negative correlation. The OTU belonging to Ascomycota dominates the interaction of the flora.

### Effects of Soil Physical and Chemical Factors on the Fungal Community

The physical and chemical factors of the root soil of different samples are shown in Table 3. According to the analysis of VIF, OM, AK, AP, AN, pH, and SM were found to be significantly related to the structure of fungal community after screening the soil physical and chemical factors, which can reflect the impact on fungal community to the greatest extent.

**TABLE 3:** Nutrient and chemical properties in the soil of the *Oxytropis glacialis* root system.

Sample	pH	Soil moisture	Electrical conductivity/(ms/cm)	Organic matte/ (mg/kg)	Available K/ (mg/kg)	Available P/ (mg/kg)	Available N/ (mg/kg)
YBJ	5.80 ± 0.06	0.143 ± 0.023	0.07 ± 0.01	145.9 ± 21.0	164.3 ± 39.3	8.91 ± 2.26	619.7 ± 18.6
ZF	7.45 ± 0.34	0.130 ± 0.007	0.11 ± 0.02	88.3 ± 7.3	134.8 ± 23.8	5.11 ± 1.06	594.9 ± 67.0
ZBY	8.82 ± 0.09	0.054 ± 0.008	0.24 ± 0.00	11.8 ± 0.2	503.9 ± 17.0	4.77 ± 2.58	320.5 ± 4.7
CQ	7.70 ± 0.09	0.091 ± 0.017	0.11 ± 0.00	102.8 ± 11.9	156.8 ± 10.6	4.59 ± 0.28	703.4 ± 96.7
VIF	34.64	8.76	48.19	20.82	35.01	2.53	7.85
Selected VIF	6.31	3.69	/	/	/	2.14	1.69

*VIF represents the VIF value of the environmental factors before selection, selected VIF represents the VIF value of the environmental factors after screening, and “-” represents the environmental factors with insignificant related effects.*

### Principal Coordinate Analysis and Canonical Correspondence Analysis

**FIGURE 5:**
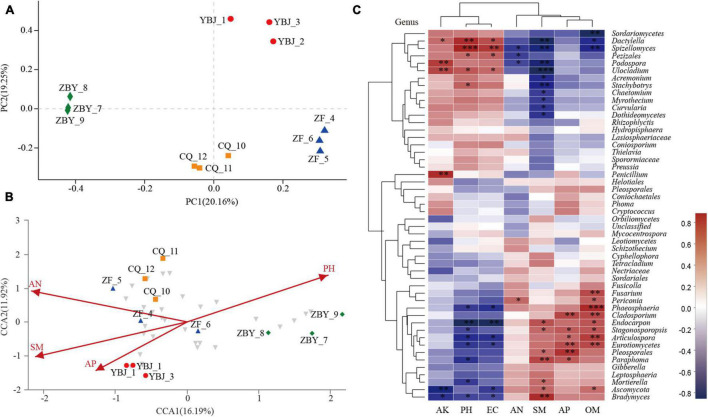
Principal coordinate analysis and Canonical correspondence analysis and correlation heatmap map at the genus level. **(A)** Principal coordinate analysis (PCoA). **(B)** Canonical correspondence analysis (CCA). **(C)** Heatmap diagram of the correlation between soil physical and chemical factors and root-soil flora based on the genus level. *R* values are shown in different colors in the figure. *P*-values less than 0.05 are marked with * (*0.01 < *P* ≤ 0.05, **0.001 < *P* ≤ 0.01, ***P ≤ 0.001).

Through principal coordinate analysis (PCoA) (Figure 5A), the first sorting axis PC1 explained 20.16%, and the second sorting axis PC2 explained 19.25%; these findings explained 39.41% of the species composition changes. The four samples showed similarities in sex and differences. While the fungal were clustered together, the CQ and ZF samples were relatively similar, and the ZBY sample was discrete from the YBJ, CQ, and ZF samples, which showed a certain degree of heterogeneity.

The CCA analysis results show that the interpretation degree of the first sorting axis (CCA1) is 16.19% (Figure 5A), and the correlation coefficients with ammonium nitrogen, available phosphorus, pH and water content are −0.9261, −0.6635, 0.8143, and −0.8943, respectively. The second sorting axis (CCA2) has an explanation degree of 11.92%, and the correlation coefficients with ammonium nitrogen, available phosphorus, pH, and water content are 0.3774, −0.7482, 0.5805, and −0.4474, respectively. In addition, the two axes explain 28.11% of the composition change in species. The fungal community was significantly correlated with pH, water content and ammonium nitrogen (*P* < 0.01) and significantly correlated with available phosphorus (*P* < 0.05). The angle between available phosphorus (Figure 5B), ammonium nitrogen and water content was acute and positively correlated, and the angle between pH was obtuse and negatively correlated. Ammonium nitrogen, available phosphorus, pH and water content determine the distribution of community species. The coefficients (*R*^2^ values) were 0.8099, 0.5544, 0.8664, and 0.8292, and the order of influence on the flora was pH > pH > SM > AP > AN.

### Heatmap of the Correlation Between the Soil Physical and Chemical Factors and the Microflora

By calculating the Spearman rank correlation coefficient between the soil physical and chemical factors and the flora, the results of the heatmap diagram (Figure 5C) show that the soil physical and chemical factors have a significant impact on the fungal community, and different fungal are affected by the soil physical and chemical factors. The study revealed that soil organic matter, available phosphorus, ammonium nitrogen, and water content were positively correlated with the fungal community, while available potassium was mainly negatively correlated with the fungal microflora. We found that 12 genera were significantly positively correlated with the soil physical and chemical factors and that 13 genera were significantly negatively correlated with the soil physical and chemical factors. Additionally, soil available potassium, pH, and conductivity were significantly positively correlated with the genera of fungal belonging to Sordariomycetes and Pezizomycetes and negatively correlated with the genera of fungal belonging to Zygomycota, Chytridiomycota, and Ascomycota. Soil ammonium nitrogen, water content, available phosphorus, and OM were significantly positively correlated with the genera of fungal in Zygomycota, Chytridiomycota, and Ascomycota and negatively correlated with the genera of fungal belonging to Sordariomycetes and Pezizomycetes. pH and *Spizellomycetaceae* were extremely positively correlated. *Spizellomycetaceae* can promote the growth of plant roots, promote the accumulation and absorption of nutrients through root tissues, and increase the nutrient and organic contents in the root soil. The water content is significantly negatively correlated with *Ulocladium*, which can assist plants in coping with drought resistance stress. OM and *Phaeosphaeria* are highly positively correlated (*P* ≤ 0.001), and the intracellular and extracellular metabolites of *Phaeosphaeri* have the function of resisting pathogenic fungal in plants through spectral analysis.

### Screening for SW-Producing Fungal

**FIGURE 6:**
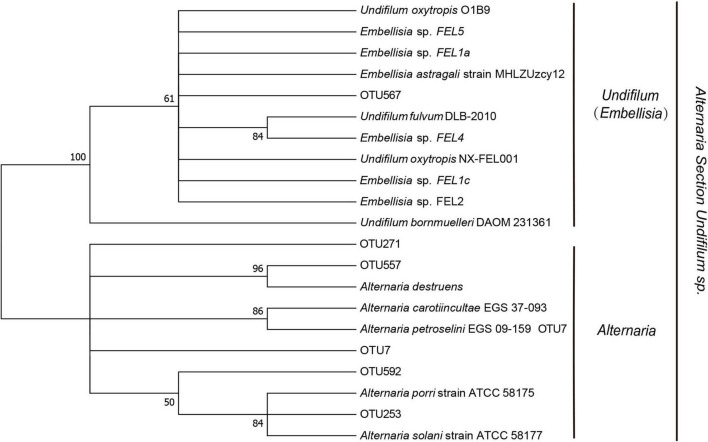
Neighbor-Joining (NJ) phylogenetic tree based on ITS sequence. The number on the evolution tree represents the self-test support rate.

There were 158 unannotated fungal in the Ascomycota, Dothideomycetes, Pleosporales, Pleosporaceae, and *Alternaria* species, where the SW fungal were located. We constructed phylogenetic treesfrom a total of six fungal sequences (Figure 6), including SW-producing fungal and known SW-producing fungal, and found that OTU7 (66.61%), OTU592 (13.05%), OTU253 (9.46%), OTU567 (7.78%), OTU557 (2.09%), and OTU271 (1.00%) had the potential to produce swainonine. Among them, OTU576 was similar to *Embellisia grisea*; OTU557, OTU7, OTU592, OTU271, and OTU253 were similar to *E. Oxytropis*; and OTU253 and OTU271 were only distributed in YBJ.

## Discussion

The regional climate type is a plateau cold zone and subcold zone monsoon semiarid climate. The population variation and distribution of soil microbes in plant roots have distinct characteristics, which are of great significance to the climate of the alpine grassland ecosystem on the QTP (Rui et al., 2015; Pérez-Jaramillo et al., 2016; Zhao et al., 2018). This study found significant differences in the soil fungal of the *O. glacialis* root system under different ecological types. Additionally, the diversity of fungal in the alpine grassland and the alpine degraded grassland was significantly higher than that in the alpine desert and alpine saline desert. In addition, locoweeds have better adaptability to this environment under extreme environmental conditions, whereas the growth of locoweed is not enough to make up for the biomass of other plants, which decreases the biomass of grassland and accelerates the degradation of alpine grassland.

The soil fungal microflora of plant roots in the same area is similar at phylum levels, and there are differences as the classification level is refined (Kowalchuk et al., 2002). In this study, as the fungal community structure was classified from the phylum to genus level during the process, the similarity of species gradually decreased, and a certain proportion of endemic genera appeared in various ecological types. Soil microbes respond to active plant selection (Chen et al., 2016). The *O. glacialis* root system is extremely developed, which helps to form a stable fungal community structure (Li, 2005). In recent years, relevant studies have been done about the diversity of soil fungal in the roots of alpine grasslands and alpine meadows and found that the dominant species are mostly distributed in Ascomycetes, Zygomycota, and Basidiomycota (Cui et al., 2019), which is similar to our research results. In addition, we annotated only ascomycetes, zygomycetes, basidiomycetes, chytridiomycetes, and Glomeromycota, while 7.29% of fungal community were unannotated, which is a great potential for the excavation of fungal resources on the QTP. Different ecological types and species that are significantly enriched lead to different fungal diversity and structural function distributions. The nerve co-occurrence network can be used to determine the core species of the flora that can maintain the diversity and structural function of the flora (Cardinale et al., 2015; Aschenbrenner et al., 2017). The 13 core fungal communities we found in this research are of great significance for the study of the adaptive evolution of locoweed such as *O. glacialis* in alpine grasslands. Moreover, it was found that the soil fungal community in the root system of *O. glacialis* increased with the classification from class to genus to OTU, and the connectivity of the microbiome-related network also became closer; thus, the fungal community tended to be more closely synergistic. For example, Ascomycota, Sordariomycetes, and Agaricomycetes have a key role in the construction of the microflora network, which reveals the adaptive evolution mechanism of fungal community on *O. glacialis* and plays an important role in extreme environments.

In previous studies, SW-producing fungal can only be isolated and identified in the roots, stems, petioles, leaves, seeds, fruit pods, and other parts of the locoweeds, and no one has conducted research on the root soil of its habitat. To study the root soil of its habitat, this study was the first to identify six OTUs with potential SW production from the root soil of *O. glacialis* by high-throughput sequencing technology. The SW-producing fungal was a pleasant surprise in our analysis of the results. OTU clustering analysis and taxonomic analysis of species based on 97% similarity using Usearch 7.0 software in the Silva database. However, SW-producing fungal were not annotated. We speculate that SW-producing fungal have been reported in recent years and are not yet included in the Silva database. We believe that SW-producing fungal are present in the soil of the *O. glacialis* root system. We re-analyzed all OTUs using MEGA7 software. Six highly similar fungal sequences were obtained by comparison with fungal sequences that have been reported to SW-producing fungal and a phylogenetic tree was constructed. With this important finding, we hope to validate it in subsequent experiments and obtain more meaningful results. This research provided evidence for the further study of the horizontal propagation of SW-producing plants. The SW-producing fungal of locoweeds have a greater ecotoxicological effect on alpine grassland ecosystems and exacerbate grassland degradation (Zhao et al., 2010). Research related to grass-produced SW fungal is in its infancy. In previous studies, gramineous plant fungal in grasslands can improve plant stress resistance such as to extreme environments, pests, diseases, cold drought, saline conditions, and alkali conditions (Nan and Li, 2004; Huang and Huang, 2008), and related studies have shown that phosphorus stress and osmotic stress stimulate seedling growth of *O. punctatus* (Cui et al., 2009). Locoweeds can grow better under low pH and drought stress in the field (Barillas et al., 2007). Locoweeds containing SW fungal reduce the number of animals that feeding on them, but the ecological effects of SW fungal on *locoweeds* have not been studied in depth.

The diversity and differential distribution of soil microbes in roots are driven by the soil physical and chemical factors (Schmidt et al., 2011; Yuan et al., 2014). Studies have shown that the soil microbial diversity in roots is mainly affected by soil pH and OM (Nacke et al., 2011). Yang et al. (2014) discovered that soil pH, temperature, ammonium nitrogen, and plant diversity are the most important factors that affect the diversity and structural function of flora. In this study, we also found that pH, water content, and ammonium nitrogen are the main factors that jointly drive the spatial variability of the fungal community in the root system of *O. glacialis*.

## Conclusion

In conclusion, the soil fungal of the *O. glacialis* root system in the different ecological environments of the QTP are rich in fungal diversity, and its core flora is stable. The spatial variation in fungal diversity is driven by pH, SM, and available N. It was also found for the first time that swainonine-producing fungal coexist in rhizosphere soil microorganisms of *O. glacialis.* This finding provides data to support the next step in demonstrating the horizontal spread of swainone-producing fungal from *O. glacialis* to soil. In addition, a stable network of core flora has a facilitating effect on the formation of *O. glacialis* as a dominant species in alpine ecosystems.

## Data Availability Statement

The datasets presented in this study can be found in online repositories. The names of the repository/repositories and accession number(s) can be found in the article/supplementary material.

## Author Contributions

P-XC drafted the manuscript. P-XC, YL, NZ, and G-QX collected the samples. P-XC, YL, and XL conceived and designed the experiments. P-XC, H-MM, NZ, and G-QX performed the experiments and data analysis. P-XC, S-TC, and XL revised the manuscript. All authors have read and approved the submitted version.

## Conflict of Interest

The authors declare that the research was conducted in the absence of any commercial or financial relationships that could be construed as a potential conflict of interest.

## Publisher’s Note

All claims expressed in this article are solely those of the authors and do not necessarily represent those of their affiliated organizations, or those of the publisher, the editors and the reviewers. Any product that may be evaluated in this article, or claim that may be made by its manufacturer, is not guaranteed or endorsed by the publisher.
